# Real life persistence rate with antimuscarinic treatment in patients with idiopathic or neurogenic overactive bladder: a prospective cohort study with solifenacin

**DOI:** 10.1186/s12894-017-0216-4

**Published:** 2017-04-13

**Authors:** Marloes J. Tijnagel, Jeroen R. Scheepe, Bertil F. M. Blok

**Affiliations:** Department of Urology, Erasmus University Medical Center, P.O. Box 2040, 3000 CA Rotterdam, The Netherlands

**Keywords:** Muscarinic antagonists, Overactive bladder, Urge urinary incontinence, Adverse effects, Medication adherence

## Abstract

**Background:**

Several studies have shown that the antimuscarinic treatment of overactive bladder is characterized by low long-term persistence rates. We have investigated the persistence of solifenacin in real life by means of telephonic interviews in a prospective cohort. We included both patients with idiopathic overactive bladder as well as neurogenic overactive bladder.

**Methods:**

From June 2009 until July 2012 patients with idiopathic or neurogenic overactive bladder who were newly prescribed solifenacin were included. In total 123 subjects were followed prospectively during one year by means of four telephonic interviews, which included questions about medication use and adverse events.

**Results:**

After one year 40% of all patients included was still using solifenacin, 50% discontinued and 10% was lost to follow-up. In the neurogenic group 58% was still using solifenacin versus 32% in the idiopathic group after one year (*p* < 0,05). The main reasons to stop solifenacin were lack of efficacy, side effects and a combination of both.

**Conclusions:**

This prospective cohort study showed a real life continuation rate of 40% after 12 months. This continuation rate is higher than found in most other studies.

The use of regular telephonic evaluation might have improved medication persistence. The findings of this study also suggest that patients with neurogenic overactive bladder have a better persistence with this method of evaluation compared to patients with idiopathic overactive bladder.

**Trial registration:**

This study was retrospectively registered on march 17, 2017 at the ISRCTN registry with study ID ISRCTN13129226.

**Electronic supplementary material:**

The online version of this article (doi:10.1186/s12894-017-0216-4) contains supplementary material, which is available to authorized users.

## Background

Antimuscarinics are the first-line therapy in the treatment of overactive bladder (OAB). This applies to idiopathic OAB (iOAB) as well as neurogenic OAB (nOAB). The use of antimuscarinics in patients with iOAB is characterized by very low persistence rates. Results from short-term studies show discontinuation rates ranging from 4 to 31% [1]. The long-term persistence to antimuscarinics in OAB is not well investigated. A systematic review conducted by Veenboer et al. found that persistence beyond 1 year rarely exceeded 10% of the patients [2]. These data might even represent an overestimation of the persistence because reviews of medical claims data show much higher discontinuation rates (up to 83% within the first 30 days) [1]. Furthermore, patients who have collected the prescribed medications might not use them because of other reasons, like fear for adverse effects.

Regarding the use of antimuscarinics in the treatment of nOAB much less studies have been performed compared to iOAB. Patients with nOAB are a heterogeneous group with different underlying neurologic conditions, such as multiple sclerosis, spinal cord injury, Parkinson disease, cerebral palsy and meningomyelocele [3]. Patients often suffer from incontinence, urgency, frequency or impaired bladder emptying. It has been shown that the use of antimuscarinics in this group is associated with better patient-reported cure/improvement compared to placebo. However, there is a higher incidence of adverse events [4].

This prospective study was carried out to investigate the persistence rate in real life among patients with idiopathic or neurogenic OAB who were prescribed solifenacin. We followed them during one year by means of telephonic interviews.

Furthermore, we wanted to investigate the reasons why patients stopped taking their medications. Third, we wanted to investigate if we could find any differences between patients with idiopathic OAB versus neurogenic OAB.

## Methods

This study was undertaken at the urology department of the Erasmus University Medical Center, Rotterdam, The Netherlands. The ethics committee of the hospital approved the study protocol. The inclusion was carried out from June 2009 until July 2012. After giving informed consent, patients older than 18 years and newly prescribed solifenacin because of complaints of idiopathic or neurogenic OAB, were included. Solifenacin, under the trade name Vesicare, is a urinary antispasmodic of the anticholinergic class. It is produced by Astellas Pharma BV. It is available in 5 and 10 mg. The starting dose was chosen by the doctor who prescribed the solifenacin but could be adjusted during the study period. Because this observational study investigated the persistence rate in real life in patients who had been prescribed solifenacin by their own doctor, they had to collect the solifenacin themselves at a pharmacy of choice.

Patients who had used anticholinergic drugs less than 7 days before they started solifenacin were excluded. Participants were allowed to continue possible other urologic medications, for example alfa-blockers, but not other anticholinergic drugs.

Telephonic surveys were taken at 1, 3, 6 and 12 months after starting solifenacin. The patients were asked whether they were continuing the medication. They were also interviewed about possible side effects and if they had discontinued the therapy, what had been reasons for stopping.

Statistical analysis was performed using SPSS statistical software. The Chi-square test was used to evaluate the differences between groups.

## Results

**Fig. 1:**
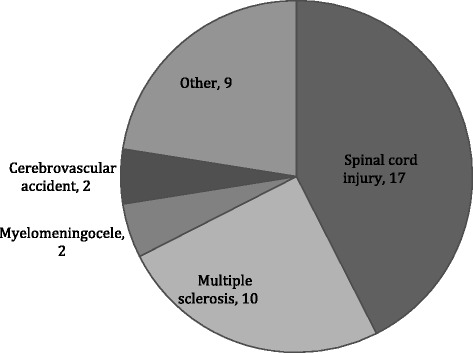
Patient distribution neurogenic OAB

During the study period a total number of 123 patients were included in this study. Twelve patients were lost to follow-up. Table 1 displays the demographic characteristics. Eighty-three patients received solifenacin because of idiopathic OAB and 40 patients because of neurogenic OAB. Among this group 17 patients had a spinal cord injury, 10 multiple sclerosis. The rest was diagnosed with other conditions as you can find in Fig. 1.

**Table 1:** Demographics

Characteristic	Patients included
Age: years	
Mean (S.D.)	61.7 (15.4)
Range	20.1 – 90.2
Gender: no (%)	
Men	70 (57%)
Women	53 (43%)
Starting dose : no (%)	
10 mg/day	12 (9.8)
5 mg/day	106 (86.2)
5 mg/2 days	3 (2.4)
2.5 mg/2 days	1 (0.8)
unknown	1 (0.8)
Condition: no (%)	
Idiopathic OAB	83 (67.5)
Neurogenic OAB	40 (32.5)

*S.D.* Standard deviation

After one year 40% of all patients included were still using solifenacin, 50% discontinued and 10% was lost to follow-up. Table 2 shows the persistence rate after one year in patients with idiopathic OAB and neurogenic OAB. Persistence in the neurogenic group was 58% versus 32% in the idiopathic group (*p* < 0,05).

**Table 2:** Persistence rate solifenacin after one year

	Patients still using	Patients discontinued	Lost to FU
All patients	50 (40.7%)	61 (49.6%)	12 (9.7%)
Neurogenic OAB	23 (57.5%)	13 (32.5%)	4 (10%)
Idiopathic OAB	27 (32.5%)	48 (57.8%)	8 (9.7%)

The main reasons to stop taking solifenacin were lack of efficacy (39%), side effects (30%) and a combination of both (13%).

Of the total group of 111 interviewed patients 64 patients (58%) experienced side effects within one year. Most common side effects were dry mouth, constipation, blurred vision, dry eyes and abdominal pain.

## Discussion

Antimuscarinic drugs have been available for many years for the treatment of OAB.

OAB is a chronic condition and long-term effective treatment might be of importance for the quality of life. Unfortunately, adherence and persistence to antimuscarinics are poor. OAB medication is known to have the lowest persistence in comparison to other chronic oral medication like cardiovascular, antidiabetic and osteoporosis treatments [5]. Regarding the use of benign prostate hyperplasia (BPH) medication, a large population-based cohort study using an administrative prescription database showed that the persistence was 29% after one year [6].

Doses of solifenacin succinate (5 mg and 10 mg) once daily (OD) have proven to be effective [7–9]. Haab et al. showed that 81% of the patients completed 40 weeks of open-label treatment with only 4,7% discontinuation because of adverse events [10]. Clinical and prescription database studies demonstrated much lower continuation rates varying from 9 to 35%. [11–15].

In our study we found a continuation rate of 40% after 12 months. This continuation rate is higher than found in most other studies. We think that this difference might be explained by the fact that the patients received telephonic interviews regularly. This is somewhat in line with other studies, which suggest that compliance to OAB therapy improves with patient education about OAB en its treatment [16, 17].

Furthermore, an additional difference in our study was the possibility of adjusting the medication dose during the study period. Patients who complained about side effects could receive a lower dose, whereas people who had little effect could receive a higher dose. This possible adjustment might have contributed to a higher persistence. This observation might encourage other caregivers to evaluate regularly patients who receive antimuscarinic medication for OAB.

A possible tool for the future is the use of Short Message Service (SMS) to improve utilization of and adherence to anticholinergic medication. It is a simple and inexpensive strategy, which has proven to help patients taking their medications on time [18]. Furthermore, it has been used to increase medication adherence to a variety of medication classes on a short term [19–22]. This tool could educate people with OAB and help them to improve persistence with antimuscarinic medication on the long term.

A large screening survey performed in the USA to identify patient-reported reasons for discontinuing overactive bladder medication found that the most mentioned reasons were: “didn’t work as expected”,” switched to new medication”, “learned to get by without medication” and “I had side effects” [23].

These reported reasons are similar to our study were the main reasons to stop taking the medications were lack of efficacy (39%), side effects (30%) and a combination of both (13%). A possible confounder of our study is that Dutch patients usually have to pay a part of the medication costs themselves when the product is still patented. No one reported these costs as a reason to stop, but we did not ask explicitly.

As mentioned before, antimuscarinic treatment in patients with neurogenic OAB has not been thoroughly evaluated. Treatment for neurogenic OAB is important in order to provide more bladder control, decrease urinary incontinence and, therefore, decrease the risk of decubitus ulcers, prevent UTI’s and ultimately to preserve renal function [24]. Antimuscarinics are advised to use as a first line medical treatment, but data on persistence in nOAB are lacking [25]. A study on the epidemiology and healthcare utilization of neurogenic bladder patients performed in the US found that 71, 5% was using one of more OAB drugs during the study period of one year. Only 29% of the patients continued that therapy. Another 38% of the patients stopped and did not restart, 34% stopped and restarted [24]. This suggests that neurogenic bladder patients are not adequately managed. In our study 32% of the patients with neurogenic OAB discontinued versus 58% of the patients with idiopathic OAB, which was a significant difference. This suggests that patients with neurogenic OAB have a better persistence compared to patients with idiopathic OAB.

## Conclusions

This prospective cohort study showed a real life continuation rate of solifenacin of 40% after 12 months. This continuation rate is higher than found in most other studies.

The use of regular telephonic evaluation might have improved medication persistence. This observation should be further investigated. The findings of this study also suggest that patients with neurogenic overactive bladder have a better persistence with this method of evaluation compared to patients with idiopathic overactive bladder.

## Additional file

Additional file 1: (Dataset 1). Real life persistence Solifenacin. This is a data set for a study of the real life persistence rate with antimuscarinic treatment in patients with idiopathic or neurogenic overactive bladder. (PDF 56 kb)

## Abbreviations

OAB: Overactive bladder
iOAB: Idiopathic overactive bladder
nOAB: Neurogenic overactive bladder
UTI: Urinary tract infection
BPH: Benign prostatic hyperplasia
SMS: Short message service

## References

[1] Sexton CC, Notte SM, Dmochowki RR, Cardozo L, Subramanian D, Coyne KS (2011). Persistence and adherence in the treatment of overactive bladder syndrome with anticholinergic therapy: a systematic review of literature. Int J Clin Pract.

[2] Veenboer PW, Bosch JL (2014). Long-term adherence to antimuscarinic therapy in everyday practice: a systematic review. J Urol.

[3] Chancellor MB, Anderson RU, Boone TB (2006). Pharmacotherapy for neurogenic detrusor overactivity. Am J Phys Med Rehabil.

[4] Madhuvrata P, Singh M, Hasafa Z, Abdel-Fattah M (2012). Anticholinergic drugs for adult neurogenic detrusor overactivity: a systematic review and meta-analysis. Eur Urol.

[5] Yeaw J, Benner JS, Walt JG, Sian S, Smith DB (2009). Comparing adherence and persistence across 6 chronic medication classes. J Manag Care Pham.

[6] Cindolo L, Pirozzi L, Fanizza C (2015). Drug adherence and clinical outcomes for patients under pharmacological therapy for lower urinary tract symptoms related to benign prostatic hyperplasia: population-based cohort study. Eur Urol.

[7] Chapple CR, Martinez-Garcia R, Selvaggi L (2005). A comparison of the efficacy and tolerability of solifenacin succinate and extended realease tolterodine at treating overactive bladder syndrome: results of the STAR trial. Eur Urol.

[8] Luo D, Liu L, Han P, Wei Q, Shen H (2012). Solifenacin for overactive bladder: a systematic review and meta-analysis. Int Urogynecol J.

[9] Cardozo L, Hessdorfer E, Milani R (2008). Solifenacin in the treatment of urgency and other symptoms of overactive bladder: results from a randomnized, double-blind, placebo-controlled, rising-dose trial. BJU Int.

[10] Haab F, Cardozo L, Chapple C, Ridder AM (2005). Long-term open-label Solifenacin treatment associated with persistence with therapy in patients with overactive bladder syndrome. Eur Urol.

[11] Yokoyama T, Koide T, Hara R, Fukumoto K, Miyaji Y, Nagai A (2013). Long-term safety and efficacy of two different antimuscarinics, imidafenacin and solifenacin, for treatment of overactive bladder: a prospective randomized controlled study. Urol Int.

[12] Brostrom S, Hallas J (2009). Persistence of antimuscarinic drug use. Eur J Clin Pharmacol.

[13] Gopal M, Haynes K, Bellamy SL, Arya LA (2008). Discontinuation rates of anticholinergic medications used for the treatment of lower urinary tract symptoms. Obstet Gynecol.

[14] Wagg A, Compion G, Fahey A, Siddiqiu E (2012). Persistence with prescribed antimuscarinic therapy for overactive bladder: a UK experience. BJU Int.

[15] Sicras-Mainar A, Rejas J, Navarro-Artieda R (2014). Antimuscarinic persistence patterns in newly treated patients with overactive bladder: a retrospective comparative analysis. Int Urolgynecol J.

[16] Brubaker L, Fanning K, Goldberg EL, et al. Predictors of discontinuing overactive bladder medications. BJU Int. 2010;105(9):1283–90. 10.1111/j.1464-410X.2009.09035.x 19912189

[17] Wyman JF, Burgio KL, Newman DK (2009). Practical aspects of lifestyle modifications and behavioral interventions in the treatment of overactive bladder and urinary incontinence. Int J Clin Prac.

[18] Huang HL, Li YCJ, Chou YC, et al. Effects of and satisfaction with short message service reminders for patient medication adherence: a randomized controlled study. BMC Med Inform Decis Mak. 2013;13:127. 10.1186/1472-6947-13-127 PMC4225681 24238397

[19] Strandbygaard U, Thomsen SF, Backer V (2010). A daily SMS reminder increases adherence to asthma treatment: a three-month follow-up study. Respir Med.

[20] Vervloet M, Van Dijk L, De Bakker DH (2014). Short- and long-term effects of real-time medication monitoring with short message service (SMS) reminders for missed doses on the refill adherence of people with Type 2 diabetes: Evidence from a randomized controlled trial. Diabet Med.

[21] Da Costa TM, Barbosa BJP, Gomes e costa DA, et al. Results of a randomized controlled trial to assess the effects of a mobile SMS-based intervention on treatment adherence in HIV/AIDS-infected Brazilian women and impressions and satisfaction with respect to incoming messages. Int J Med Inform. 2012;81(4):257–69. 10.1016/j.ijmedinf.2011.10.002 PMC3766367 22296762

[22] Wang K, Wang C, Xi L (2014). A randomized controlled trial to asses adherence to allergic rhinitis treatment following a daily short message service (SMS) via the mobile phone. Int Arch Allergy Immunol.

[23] Benner JS, Nichol MB, Rovner ES (2009). Patient-reported reasons for discontinuing overactive bladder medication. BJU Int.

[24] Manack A, Motsko SP, Haag-Molkenteller C (2011). Epidemiology and healthcare utilization of neurogenic bladder patients in a US claims database. Neurourol Urodyn.

[25] Groen J, Pannek J, Castro Diaz D (2016). Summary of European Association of Urology (EAU) Guidelines on Neuro-Urology. Eur Urol.

