# Supplementary material for: Real life persistence rate with antimuscarinic treatment in patients with idiopathic or neurogenic overactive bladder: a prospective cohort study with solifenacin
Source: BMC Urol. 2017 Apr 13;17:30. doi: 10.1186/s12894-017-0216-4 (PMC5390358; doi:10.1186/s12894-017-0216-4)
Supplement: Additional file 1: (Dataset 1). — Real life persistence Solifenacin. This is a data set for a study of the real life persistence rate with antimuscarinic treatment in patients with idiopathic or neurogenic overactive bladder. (PDF 56 kb) [file 12894_2017_216_MOESM1_ESM.pdf]

Dataset 1. Real life persistence Solifenacin

This is a data set for a study of the real life persistence rate with antimuscarinic treatment in patients with idiopathic or neurogenic overactive bladder

corresponding author: M. Tijnagel (mtijn@hotmail.com)
Please contact M. Tijnagel if you plan to conduct analyses on these data for publication

variable	description
sex	
age	
starting dose	starting dose of solifenacin
condition	idiopathic of neurogenic overactive bladder
still taking	persistence after one year (J= Yes,N=No)
why stop	reason mentioned why participant had stopped taking solifenacin
side effects	presence of side effects observed by the participant (J= Yes,N=No)
type of side effect	type of side effect as mentioned by participant

Sex	Age	starting dose	Condition	still taking?	Why stop	Side effects	Type of side effect
F	70-74	10 mg/day	I	J		J	dry mouth
F	80-84	5 mg/day	I	J		J	dry mouth
F	75-79	5 mg/day	I	J		J	dry mouth
F	75-79	5 mg/day	I	J		J	dry mouth
F	50-54	5 mg/day	I	J		J	dry mouth
F	65-69	5 mg/day	I	J		J	constipation
F	70-74	5 mg/day	I	J		J	blurred vision, constipation, dry mouth
F	85-89	5 mg/day	I	J		N	
F	60-64	5 mg/day	I	J		N	
F	45-49	5 mg/day	N	J		J	edema
F	45-49	5 mg/day	N	J		J	dry eyes
F	55-59	10 mg/day	N	J		J	fatique
F	60-64	5 mg/day	N	J		N	
F	60-64	5 mg/day	N	J		N	
F	55-59	5 mg/day	N	J		N	
F	55-59	5 mg/day	N	J		N	
F	45-49	5 mg/day	N	J		N	
F	40-44	5 mg/day	N	J		N	
F	40-44	5 mg/day	N	J		N	
F	30-34	5 mg/day	N	J		N	
M	60-64	10 mg/day	I	J		J	less diarhea
M	80-84	10 mg/day	I	J		J	dry mouth
M	80-84	5 mg/day	I	J		J	dry mouth
M	75-79	5 mg/day	I	J		J	dry mouth
M	70-74	5 mg/day	I	J		J	dry mouth
M	60-64	5 mg/day	I	J		J	dry mouth
M	60-64	5 mg/day	I	J		J	dry mouth
M	35-39	5 mg/ day	I	J		J	heartburn, constipation

M	90-94	10 mg/day	I	J		N	
M	55-59	10 mg/day	I	J		N	
M	75-79	2,5 mg/2 days	I	J		N	
M	80-84	5 mg/day	I	J		N	
M	80-84	5 mg/day	I	J		N	
M	70-79	5 mg/day	I	J		N	
M	75-79	5 mg/day	I	J		N	
M	65-69	5 mg/day	I	J		N	
M	55-59	5 mg/day	I	J		N	
M	45-49	5 mg/day	I	J		N	
M	55-59	5 mg/day	N	J		J	dry skin, itch, dry mouth
M	45-49	5 mg/day	N	J		J	dry mouth
M	40-44	5 mg/day	N	J		J	dry mouth
M	30-34	5 mg/day	N	J		J	dry mouth
M	70-74	5 mg/day	N	J		J	dry mouth, constipation
M	60-64	10 mg/day	N	J		J	dry mouth
M	50-54	5 mg/day	N	J		J	constipation, dry mouth
M	40-44	10 mg/day	N	J		N	
M	25-29	10 mg/day	N	J		N	
M	65-69	5 mg/day	N	J		N	
M	60-64	5 mg/day	N	J		N	
M	50-54	5 mg/day	N	J		N	
F	65-69	5 mg/day	I	N	side effects	J	abdominal pain, dty mouth, constipation, nausea
F	60-64	5 mg/day	I	N	lack of efficacy	J	abdominal pain, dry mouth, constipation, blurred vision
F	65-69	10 mg/day	I	N	lack of efficacy	J	dry mouth
F	85-89	5 mg/2 days	I	N	unknown	J	dry mouth
F	65-69	5 mg/day	I	N	lack of efficacy and side effects	J	dry mouth
F	45-49	5 mg/day	I	N	lack of efficacy and side effects	J	dry mouth
F	40-44	5 mg/day	I	N	lack of efficacy	J	dry mouth
F	45-49	5 mg/day	I	N	unknown	J	dry mouth, constipation, blurred vision, abdominal pain

F	55-59	5 mg/day	I	N	side effects	J	dry mouth, nausea, dizziness
F	65-69	5 mg/day	I	N	side effects	J	dry mouth, constipation, headache
F	50-54	5 mg/day	I	N	side effects	J	dry eyes, dry mouth
F	75-79	5 mg/day	I	N	side effects	J	palpitations
F	60-64	5 mg/2 days	I	N	side effects	J	headache
F	20-24	5 mg/day	I	N	side effects	J	stomach pain
F	80-84	5 mg/day	I	N	side effects	J	fatique, dizzines
F	60-64	5 mg/day	I	N	side effects	J	edema
F	40-44	5 mg/day	I	N	lack of efficacy and side effects	J	abdominal pain
F	65-69	5 mg/day	I	N	residu	J	residu
F	60-64	5 mg/day	I	N	lack of efficacy and side effects	J	blurred vision, edema
F	50-54	5 mg/day	I	N	lack of efficacy	J	blurred vision, dry mouth
F	45-49	5 mg/day	I	N	lack of efficacy	N	
F	45-49	5 mg/day	I	N	lack of efficacy		
F	50-54	5 mg/day	N	N	side effects	J	concentration problems, constipation
F	60-64	5 mg/day	N	N	lack of efficacy	J	dry mouth
F	55-59	5 mg/day	N	N	lack of efficacy	J	dry mouth
F	50-54	5 mg/day	N	N	side effects	J	dry mouth
F	40-44	5 mg/day	N	N	lack of efficacy	N	
F	20-24	5 mg/day	N	N	lack of efficacy	N	
M	70-74	5 mg/day	I	N	unknown	J	constipation
M	70-74	5 mg/day	I	N	lack of efficacy	J	dry mouth
M	65-69	5 mg/day	I	N	lack of efficacy	J	dry mouth
M	65-69	5 mg/day	I	N	lack of efficacy	J	dry mouth, constipation
M	75-79	5 mg/day	I	N	unknown	J	dry mouth, constipation
M	75-79	5 mg/day	I	N	lack of efficacy and side effects	J	dry mouth, hesitation
M	65-69	10 mg/day	I	N	lack of efficacy and side effects	J	dry mouth, constipation, dry eyes, blurred vison
M	70-74	5 mg/day	I	N	side effects	J	dry eyes, stomach pain
M	70-74	5 mg/day	I	N	side effects	J	glaucoma, red eyes
M	75-79	5 mg/day	I	N	lack of efficacy and side effects	J	constipation

M	70-74	5 mg/day	I	N	side effects	J	constipation, dry mouth
M	65-69	5 mg/day	I	N	residu	J	residu
M	80-84	5 mg/day	I	N	lack of efficacy	N	
M	80-84	5 mg/day	I	N	ran out of medication	N	
M	80-84	5 mg/day	I	N	lack of efficacy	N	
M	75-79	5 mg/day	I	N	unknown	N	
M	70-74	5 mg/day	I	N	lack of efficacy	N	
M	70-74	5 mg/day	I	N	lack of efficacy	N	
M	70-74	5 mg/day	I	N	lack of efficacy	N	
M	65-69	5 mg/day	I	N	not allowed to used because of other medi	N	
M	65-69	5 mg/day	I	N	lack of efficacy	N	
M	55-59	5 mg/day	I	N	lack of efficacy	N	
M	45-49	5 mg/day	I	N	lack of efficacy	N	
M	45-49	5 mg/day	I	N	complaints dysappeared	N	
M	20-24	5 mg/day	I	N	lack of efficacy	N	
M	75-79	5 mg/day	I	N	unknown		
M	70-74	5 mg/day	N	N	unknown	J	constipation
M	60-64	5 mg/day	N	N	side effects	J	droge mond en maagpijn
M	60-64	5 mg/day	N	N	side effects	J	itch
M	70-74	5 mg/day	N	N	not allowed to used because of other medi	N	
M	50-54	5 mg/day	N	N	lack of efficacy and side effects	N	
M	40-44	5 mg/day	N	N	lack of efficacy	N	
M	35-39	5 mg/day	N	N	lack of efficacy	N	
F	missing	missing	I	unknown		J	constipation
F	75-79	5 mg/2 days	I	unknown			
F	missing	5 mg/day	I	unknown			
F	45-49	5 mg/day	N	unknown			
F	missing	5 mg/day	N	unknown			
M	65-69	5 mg/day	I	unknown		N	
M	45-49	5 mg/day	I	unknown		N	

M	80-84	5 mg/day	I	unknown			
M	70-74	5 mg/day	I	unknown			
M	70-74	5 mg/day	I	unknown			
M	20-24	10 mg/day	N	unknown			
M	50-54	5 mg/day	N	unknown			
